# Membrane Compartment Occupied by Can1 (MCC) and Eisosome Subdomains of the Fungal Plasma Membrane

**DOI:** 10.3390/membranes1040394

**Published:** 2011-12-13

**Authors:** Lois M. Douglas, Hong X. Wang, Lifang Li, James B. Konopka

**Affiliations:** 1 Department of Molecular Genetics and Microbiology, Stony Brook University, Stony Brook, NY 11794-5222, USA; E-Mails: lmardouglas@gmail.com (L.M.D.); hxwang@hotmail.com (H.X.W.); lifli@ic.sunysb.edu (L.L.); 2 Graduate Program in Genetics, Stony Brook University, Stony Brook, NY 11794-5222, USA

**Keywords:** MCC, eisosome, Pil1, Lsp1, Sur7, Nce102, plasma membrane, cell wall, yeast, hyphae

## Abstract

Studies on the budding yeast *Saccharomyces cerevisiae* have revealed that fungal plasma membranes are organized into different subdomains. One new domain termed MCC/eisosomes consists of stable punctate patches that are distinct from lipid rafts. The MCC/eisosome domains correspond to furrows in the plasma membrane that are about 300 nm long and 50 nm deep. The MCC portion includes integral membrane proteins, such as the tetraspanners Sur7 and Nce102. The adjacent eisosome includes proteins that are peripherally associated with the membrane, including the BAR domains proteins Pil1 and Lsp1 that are thought to promote membrane curvature. Genetic analysis of the MCC/eisosome components indicates these domains broadly affect overall plasma membrane organization. The mechanisms regulating the formation of MCC/eisosomes in model organisms will be reviewed as well as the role of these plasma membrane domains in fungal pathogenesis and response to antifungal drugs.

## 1. Introduction

The plasma membrane (PM) plays critical roles in cellular regulation in addition to acting as an important barrier. It mediates a wide range of essential processes including environmental sensing, nutrient uptake, cellular morphogenesis, secretion, and cell wall biogenesis. The importance of the PM is highlighted by the fact that most pharmaceutical drugs, including the most commonly used antifungal drugs, target PM components [1]. Unfortunately, PM organization is poorly understood due to the technical difficulties in studying hydrophobic membranes. Because of these limitations, many aspects of PM organization, such as the structure and function of lipid rafts, remain controversial [2]. However, recent studies have demonstrated that the fungal PM consists of at least three distinct subdomains that appear to have specialized functions. One domain consists of a series of immobile 300 nm-sized patches that were named Membrane Compartment occupied by Can1 (MCC) to describe the punctate localization of the Can1 arginine permease [3,4,5]. The MCC domains were subsequently shown to be associated with peripheral membrane proteins that form a complex on the inner surface of the PM called the eisosome [6]. Another domain termed Membrane Compartment occupied by Pma1 (MCP) is named for the presence of the plasma membrane ATPase Pma1 and is defined as the PM regions containing readily diffusible proteins that are excluded from the MCC [5]. A third domain consists of punctate patches containing the TORC2 complex that regulates cell polarity and ceramide synthesis [7]. Other specialized PM domains are also likely to exist, such as the PM region at hyphal tips that was identified as having an altered lipid environment, because it stains more readily with the ergosterol-binding drug filipin [8].

MCC/eisosomes will be the subject of this review, because recent studies have begun to reveal how these domains are assembled and how they contribute to PM organization and function. Most of the review will focus on MCC/eisosomes in the yeast *S. cerevisiae*, since they were first discovered and have been examined in the most detail in this organism. These studies have shown that MCC/eisosomes are associated with membrane invaginations that appear as 50 nm deep furrows [9] that are distinct from the mobile cortical actin patches and membrane invaginations detected at sites of endocytosis [4,10]. The analysis of MCC/eisosomes in other model fungi will then be summarized, as they have been detected in diverse fungi including *Ashbya gossypii* [11], *Aspergillus nidulans* [12], and *Schizzosaccharomyces pombe* [13,14]. Emphasis will be given to the distinct properties of MCC/eisosomes in other species, such as the presence of longer eisosomes in *S. pombe* [13]. Finally, the studies on MCC/eisosomes in the human fungal pathogen *Candida albicans* will be reviewed, with an emphasis on their role in regulation of cell morphogenesis and production of virulence factors.

## 2. MCC/Eisosome Domains in *S. Cerevisiae*

### 2.1. Organization of MCC/Eisosomes

**Figure 1:**
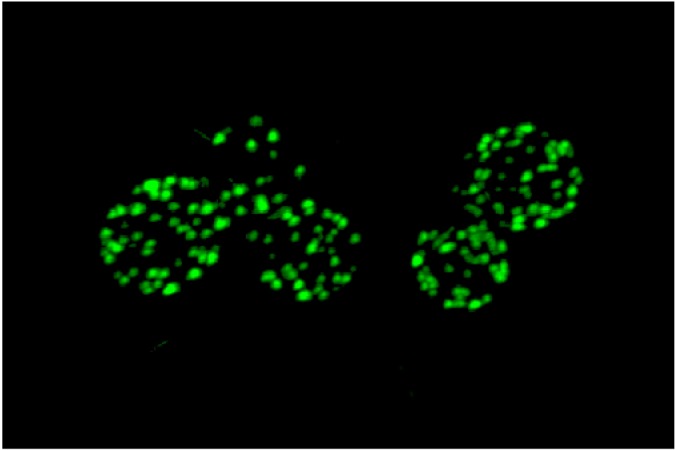
Punctate localization of Sur7-GFP in *S. cerevisiae*. Deconvolution microscopy was used to create a three dimensional reconstruction of Sur7-GFP localization.

**Figure 2:**
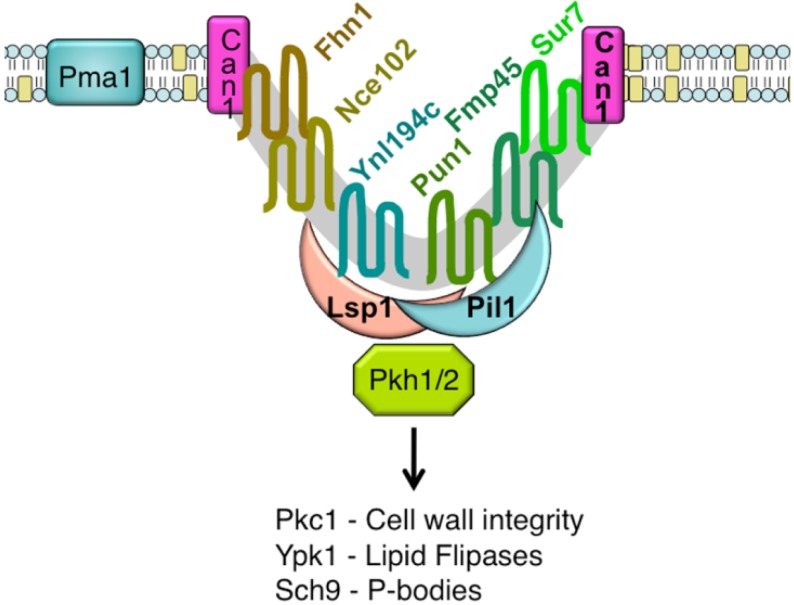
Model for MCC/eisosome structure.

An unusual punctate pattern of PM localization was first described for Sur7-GFP in *S. cerevisiae* [10], and is shown in Figure 1. This localization was surprising because the *SUR7* gene was identified as a high copy suppressor of an endocytosis defective *rvs161* strain, yet the Sur7-GFP patches did not colocalize with actin patches at sites of endocytosis. Independent studies discovered that a GFP fusion to the Can1 arginine permease also localized to punctate patches and defined these sites as a unique PM domain that was termed MCC [3,4,5]. Subsequent studies identified other integral membrane protein constituents of MCCs, including several nutrient symporters and two different families of proteins that are predicted to contain four membrane-spanning domains (tetraspanners) [15,16,17]. One family of tetraspanners found in MCCs includes Sur7 and the paralogous proteins Fmp45, Pun1, and Ynl194c. The Sur7 proteins stably associate with the MCC. The other family of tetraspanners is comprised of Nce102 and Fhn1. Interestingly, Nce102 can be detected outside the MCC, but moves into these domains in response to sphingolipids [16]. Transporters for uracil (Fur4) and tryptophan (Tat2) are also enriched in the MCC. The nutrient transporters appear to be able to move in and out of MCC domains, and their patchy localization can be more easily disrupted by mutations or loss of membrane potential [16,18,19]. Once formed, MCC and eisosomes appear to be very stable and immobile in the PM. One study suggested that MCC/eisosomes were stabilized by connection to the cell wall [10]; however, further analysis did not find evidence for cell wall attachment [4]. The MCC proteins are listed in Table 1 and a model for MCC/eisosome organization is diagrammed in Figure 2.

**Table 1:** *S. cerevisiae* MCC proteins.

**Protein**	**ORF**	**Function**	**Localization reference**	**Copies/cell 1**
Sur7	YML052W	Sur7 family tetraspanner	[3,10]	17,000
Fmp45	YDL222C	Sur7 family tetraspanner	[10]	329
Pun1	YLR414C	Sur7 family tetraspanner	[15]	1,660
Ynl194c	YNL194C	Sur7 family tetraspanner	[10]	ND
Nce102	YPR149W	Nce102 family tetraspanner	[15]	ND
Fhn1	YGR131W	Nce102 family tetraspanner	[15]	ND
Can1	YEL063C	H^+^-driven arginine permease	[3]	ND
Fur4	YBR021W	H^+^-driven uracil permease	[4]	ND
Tat2	YOL020W	H^+^-driven tryptophan and tyrosine permease	[19]	752

1 Copies of proteins per cell from data reported by [20]. ND indicates no data available.

Pil1 and Lsp1 were subsequently found to be peripheral membrane proteins that form punctate clusters termed eisosomes on the cytoplasmic surface of the PM at MCC sites [6]. As will be described below, several lines of evidence indicate that Pil1 and Lsp1 play key roles in forming MCC/eisosomes. These proteins associate with the PM via their BAR domains (named for the Bin/Amphiphysin/Rvs proteins) [21], which bind membranes and promote curvature. At least 17 other proteins are now thought to localize to eisosomes. They were identified using various approaches, including protein interaction studies and high throughput analysis of the subcellular localization of GFP-tagged proteins [15,16,22,23,24,25]. The protein components of eisosomes and their relative abundance in the cell are listed in Table 2. Some of the significant proteins present in eisosomes are Eis1, Seg1/Ymr086w, the Pkh1 and Pkh2 protein kinases, and the Slm1 and Slm2 proteins [11,24,26,27,28]. The Slm proteins are interesting in that they are also predicted to contain a type of BAR domain [29]. As described below, mutations of these proteins affect eisosome formation or stability and the Pkh1/2 kinases are also known to regulate a wide range of other cell functions [26,27,30]. Thus, it seems likely that eisosome stability can be attributed to the concerted action of multiple proteins in these complexes.

In addition to being enriched in a special set of proteins, the PM at sites of MCC/eisosomes is also thought to contain a special lipid composition. The ergosterol binding agent filipin preferentially stains MCC domains, indicating that these domains are either enriched in ergosterol or that the lipid composition is altered in a way that makes ergosterol more accessible [19].

A major breakthrough in the understanding of MCC/eisosome structure resulted from high-resolution TEM studies, which showed that these domains occur at sites of invaginations in the PM [9]. These membrane invaginations are in the shape of furrows that are about 300 nm long and 50 nm deep. Similar PM furrows have been described in a number of fungi, but their significance was not known other than that they were distinct from the finger-like projections that occur at sites of endocytosis [31,32,33,34,35]. Interestingly, immuno-EM studies indicate that Sur7 is localized at the upper edges of the furrows, whereas Pil1 is localized at the bottom of furrows [9]. Since there are about 17,000 copies of Sur7 per cell [20], and assuming an average membrane-spanning α-helix is approximately 1 nm in diameter, there is likely to be enough Sur7 to form a ring around the perimeter of each of the ∼40 furrows in a typical cell. Similar approximations indicate that there is enough Pil1 and Lsp1 to coat the inner surface of the furrows. It is estimated that there are about 10^5^ copies each of Pil1 and Lsp1 per cell [20], and assuming these proteins have a footprint on the PM of at least 10 nm^2^ [29,36], then there should be enough Pil1 and Lsp1 per cell to cover the inner surface of the furrows. Other eisosome components are thought to be less abundant, and would not add much to the area of the furrows covered by protein. Thus, these estimates of protein abundance fit with expectations based on the patterns of protein localization detected by immuno-EM.

**Table 2:** *S. cerevisiae* eisosome proteins.

**Protein**	**ORF**	**Function**	**Localization reference**	**Copies/cell 1**
Pil1	YGR086C	BAR domain	[6]	115,000
Lsp1	YPL004C	BAR domain	[6]	104,000
Pkh1	YDR490C	Ser/Thr protein kinase	[26,37]	ND
Pkh2	YOL100W	Ser/Thr protein kinase	[26,37]	ND
Eis1	YMR031C	Unknown	[15]	5,570
Slm1	YIL105C	BAR domain and PH domain	[15,38]	5,190
Slm2	YNL047C	BAR domain and PH domain	[15,38]	2,610
Seg1	YMR086w	Unknown	[25,39]	ND
Mdg1	YNL173C	Unknown	[15]	1,240
Pst2	YDR032C	Similar to flavodoxin-like proteins	[15]	2,330
Rfs1	YBR052C	Similar to flavodoxin-like proteins	[15]	7,060
Ycp4	YCR004C	Similar to flavodoxin-like proteins	[15]	14,600
Ygr130c	YGR130c	Unknown	[15,25]	10,300
Rgc1	YPR115w	Pleckstrin homology domain containing protein	[39]	300
Aim3	YBR108w	Unknown	[39]	ND
Mrp8	YKL142w	Unknown	[23,40]	1,550
Sap1	YER047c	Putative ATPase of the AAA family	[23]	300
Yta6	YPL074w	Putative AAA family ATPase	[22]	259
Msc3	YLR219w	Protein of unknown function	[23,41]	131

1 Copies of proteins per cell from data reported by [20]. ND indicates no data available.

### 2.2. Regulation of MCC/Eisosome Assembly and Disassembly

Deletion mutant analysis indicates that Pil1 plays an essential role in promoting MCC/eisosome formation [6,15,24,42]. Other MCC and eisosome proteins fail to localize properly in a *pil1Δ* mutant. Although Pil1 and Lsp1 are highly related paralogs, Lsp1 binds the PM less efficiently and is not functionally equivalent to Pil1 [6,29]. *lsp1Δ* cells appear to produce normal MCC/eisosomes. In contrast, the *pil1Δ* cells typically form one or two large complexes of the remaining MCC/eisosome proteins that are sometimes referred to as remnants. Bioinformatic predictions and the crystal structure of Lsp1 indicated that Pil1 and Lsp1 are structurally related to BAR domain proteins that bind membranes and promote curvature [29,36]. This was verified by showing that Pil1 promotes tubulation of vesicles *in vitro* and forms punctate patches when produced in mammalian cells [29,36]. Mutation of the basic residues in Pil1 that are predicted to interact with membranes diminished its ability to bind membranes and decreased the number of eisosomes that formed. However, the eisosomes that did form developed at a normal rate [29]. Thus, it was proposed that membrane association of Pil1 plays a critical role in initiating eisosome formation. Consistent with this, gene dosage studies indicated that Pil1 is a limiting component for the number and size of eisosomes [41]. Purified Pil1 can also form filaments that associate with membranes [29], indicating that Pil1 is likely to be sufficient to promote eisosome formation in yeast.

The Pil1 and Lsp1 proteins are phosphorylated by Pkh1/2 protein kinases, which are known to be activated by changes in sphingolipid levels [26,27]. The modification of Pil1 and Lsp1 by phosphorylation appears to regulate MCC/eisosomes; however, whether phosphorylation promotes MCC/eisosome assembly or disassembly is controversial. In one model, increased sphingolipid levels were proposed to promote eisosome formation by stimulating Nce102 to enter MCC domains where it would prevent the Pkh1/2 kinases from phosphorylating Pil1 and Lsp1 [16]. In this model, phosphorylation of Pil1 and Lsp1 promotes eisosome disassembly. Consistent with this, many of the phosphorylation sites reside on the predicted membrane-binding surface of Pil1/Lsp1 where the negatively charged phosphate groups could interfere with membrane association. However, other studies have proposed a model that increased phosphorylation of Pil1 and Lsp1 promotes eisosome assembly or stability [27]. In this model, increased sphingolipids are thought to stimulate the Pkh kinases to phosphorylate Pil1/Lsp1 and result in more and bigger eisosomes. A complicating factor in comparing these studies on the role of Pil1 phosphorylation is that the different research groups mutated similar, but not identical sets of phosphorylation sites [16,26,27]. It is therefore interesting that an alternative approach of using an inhibitor of the Pkh1/2 kinases to block phosphorylation of Pil1/Lsp1, rather than mutating phosphorylation sites, supported the conclusion that phosphorylation is needed for eisosome assembly or stability [43].

A recent proteomic study of *C. albicans* also raised the possibility that Pil1 and Lsp1 are regulated by SUMO, an ubiquitin-like post-translational modification [44]. Pil1 was found to be modified by attachment of SUMO to the Lys-63 residue, which is thought to be on the surface of Pil1 that faces the PM and could therefore have an effect membrane association [29,36]. Lys-63 is highly conserved in Pil1 and Lsp1 proteins from other species, further suggesting that this residue is functionally important.

Additional genes that influence MCC/eisosomes formation or stability were identified in large scale genetic screens of *S. cerevisiae* mutants [15,16,24]. Most mutations decreased eisosome size or number, and affected genes involved in lipid synthesis, vesicle trafficking, and actin organization. Several genes that were required for proper eisosome formation were found to encode proteins that localize to eisosomes including Eis1/Ymr031c, Seg1/Ymr086w, and the Slm1/2 proteins [11,15,24,28]. Deletion of *EIS1* caused increased cytoplasmic localization of Pil1. The deletion of *SEG1/YMR086W* has not yet been reported in *S. cerevisiae*, but deletion of this gene in the related fungus *A. gossypii* caused a defect in eisosome stability [11]. The Slm1/2 proteins are also needed for proper eisosomes; their mutation resulted in fewer eisosomes and accumulation of Pil1 in the cytoplasm [28]. Interestingly, the Slm1/2 proteins are also predicted to contain a type of BAR domain that can associate with membranes and promote curvature [29]. Eis1, Seg1, and Slm1/2 are less abundant than Pil1 or Lsp1, suggesting that they have a supporting role and are not major components of eisosomes (Table 2).

There must also be additional mechanisms that regulate the size and spatial location of MCC/eisosomes. For example, MCC/eisosome formation is restricted to growing buds, as new MCC/eisosomes do not form in the mother cell [41]. Restriction of eisosome formation to the buds is likely due at least in part to the regulation of *PIL1* and *LSP1* expression to the early part of the cell cycle when buds are forming [45]. There must also be a process that ensures that MCC/eisosomes are spatially distributed since they do not touch or overlap. Recent studies also indicate that MCC/eisosome size is regulated by cellular factors and not just by the availability of Pil1 and Lsp1. A mutation that increases eisosome length was detected in *S. cerevisiae*, but it is not yet clear if it affects *MAK3*, an N-terminal acetyltransferase, or the adjacent *YPR050C* gene [9]. Dramatic evidence for the influence of cellular factors on eisosome size also comes from studies on the fission yeast *S. pombe*, as will be described in more detail below [14].

### 2.3. Functional Roles of MCC/Eisosomes

Mutation of MCC/eisosome genes has indicated that these PM subdomains are needed for proper cellular organization and efficient response to a wide range of stresses. Pil1 and Lsp1 are also needed for efficient endocytosis, which led to the controversial proposal that eisosomes are sites of an alternative type of endocytic pathway [6]. A role for MCC/eisosomes in endocytosis was first suggested by the observation that *SUR7* overexpression suppressed the growth defects of an *rvs167* endocytosis mutant in *S. cerevisiae* [46]. The Pkh1/2 proteins are also needed for endocytosis [26,27,47]. Sites of active endocytosis were localized to MCC/eisosomes in one study [6], but it is not clear how they relate to the sites of endocytosis mediated by actin patches in the PM. There does not appear to be any overlap between sites of actin-mediated endocytosis and eisosomes in several different organisms that have been analyzed [6,10,11,12,13,48]. However, it is clear that mutation of *PIL1* causes a defect in endocytosis that is due at least in part to a failure to recruit key components of the endocytosis machinery to the endocytic sites [49].

Other studies have suggested an opposite role for MCC/eisosomes: that they act as protected islands to prevent endocytosis of PM proteins [15]. Part of the evidence for this was that Can1-GFP appeared to be more stable at the PM when in eisosomes than when it was induced to leave MCC/eisosomes by addition of its substrate arginine [15]. However, other studies have shown that Can1-GFP exchanges quickly between the MCC and the MCP, so that the overall rate of Can1-GFP endocytosis is not obviously affected by association with the MCC [18].

MCC/eisosome domains have also been suggested to be involved in the response to osmotic shock and dehydration. *S. cerevisiae* cells exposed to hyperosmotic conditions displayed alterations in the PM, including invaginations, which presumably help to accommodate the rapid decrease in cell volume. Under some conditions there was lateral reorganization of the PM as evidenced by redistribution of Sur7-GFP, suggesting that MCC/eisosome reorganization is part of the process by which cells resist hyperosmotic conditions [50].

Mutation of other MCC/eisosome components also affects other stress responses. Deletion of *SUR7* causes defects in sphingolipid composition, sporulation and osmotic stress [10,51]. Other Sur7 family members (Fmp45, Ynl194c, and Pun1) are implicated in nitrogen stress, cell wall integrity, and survival in stationary phase [52,53,54]. The Pkh1/2 kinases regulate other important protein kinases including Pkc1 Sch9 and Ypk1 and influence cell wall integrity, actin localization, and response to heat stress [26,27,30]. Interestingly, signaling of Pkh1/2 through Pkc1 has recently been reported to regulate deadenylation-dependent mRNA decay in *S. cerevisiae* [55]. Regulation of global mRNA degradation in this manner is an essential process that is important for controlling eukaryotic gene expression. In addition, the Pkh1/2-Pkc1 pathway mediates assembly of cytoplasmic P-body complexes that repress and store mRNA. Since both of these mRNA regulatory events occur only under nutrient-depleted conditions, Pkh1/2-Pkc1 signaling may coordinate mRNA decay and storage with other cellular mechanisms that monitor nutrient availability and other environmental stresses [55].

An interesting speculation is that MCC/eisosomes may also function to organize proteins with similar proton flux activities. This was suggested by the discovery that proton symporters for arginine, uracil and tryptophan localize to MCC/eisosomes in *S. cerevisiae* [3,4,19]. Segregation of the inward proton flux of the symporters away from the outward proton pumping activity of the plasma membrane H^+^ATPase Pma1, which is restricted from entering the MCC, may therefore have important physiological significance [3,19]. The proton symporters are distinct from Sur7 in that their presence in MCC/eisosomes is dependent on the membrane potential [19]. However, it is not clear that all proton symporters are present in the MCC and disruption of MCC domains does not prevent function of Can1 Arginine symporters [19].

## 3. Comparative Analysis of MCC/Eisosomes in Other Model Organism Fungi

### 3.1. Ashbya gossypii

Although *A. gossypii* grows as a filamentous fungus, it is actually much more closely related phytogenetically to the budding yeast *S. cerevisiae* than it is to other filamentous fungi. As expected, *A. gossypii* eisosomes bear many similarities to those in *S. cerevisiae*, including the localization of Pil1 and Lsp1 in a punctate pattern in the PM [11]. The *A. gossypii* eisosomes are also very stable and they form in a subapical region of new growth that is comparable to the region of new growth where eisosomes form in *S. cerevisiae* buds. Similar to *S. cerevisiae*, Lsp1 did not localize properly in the absence of Pil1, and Lsp1 was not required for Pil1 to localize to eisosomes. Interestingly, the main zone of endocytosis at hyphal tips lacks eisosomes.

There are also many significant differences between eisosomes from *A. gossypii* and *S. cerevisiae*. One major difference between these organisms is that there was no obvious effect on eisosome formation caused by deletion of the only apparent *NCE102* ortholog in *A. gossypii* [11]. Another difference is that an *A. gossypii pil1Δ* mutant displayed strong defects in polarized growth: abnormal morphogenesis resulted in misshapen hyphal tips, and polar surface expansion was reduced. In addition, some of the paralogous gene pairs present in *S. cerevisiae eisosomes* (Slm1 and Slm2, Pkh1 and Pkh2, and Nce102 and Fhn1) are encoded by only one gene in *A. gossypii* [11].

Eisosome stability was decreased in *A. gossypii* by deletion of *SEG1* (ABL037c), which encodes a protein that localizes to eisosomes [11]. The Seg1 protein was concluded to function in eisosome stability rather than formation. This was because the *seg1Δ* mutant was capable of forming eisosomes at growing hyphal tips, but then up to 90% of the newly formed eisosomes disassembled, concomitant with the formation of aggregates of eisosome components. The *S. cerevisiae* ortholog of Seg1, Ymr086w, has not been studied extensively, but it copurifies with eisosome proteins [25,39].

### 3.2. Aspergillus nidulans

*A. nidulans* grows as a filamentous fungus, and it can also form asexual spores called conidiospores. Consistent with *A. nidulans* being much more distantly related to *S. cerevisiae* than is *A. gossypii*, there were many differences in MCC/eisosomes. For one, the two orthologs of Pil1 present in *A. nidulans* (PilA and PilB) appear to have arisen from a duplication event that is distinct from the evolution of Pil1 and Lsp1 in *S. cerevisiae* [12]. Fluorescent protein fusions to PilA, PilB, and the Sur7 ortholog (SurG) colocalized at the cell cortex of conidiospores in punctate structures. However, the punctate patches were not as clearly distinct from each other as the eisosome patches observed in *S. cerevisiae*. In hyphae, PilA-GFP was present in punctate patches, but the patches were not uniform in size and not restricted to the periphery. PilB-GFP appeared to localize in a diffuse pattern in the cytoplasm and SurG-GFP was detected in the vacuole and endosomes. Deletion of *PILA*, *PILB*, or *SURG* did not lead to any obvious growth phenotype, except for moderate resistance to itraconazole [12]. No obvious association was found between the eisosome-like structures and sites of endocytosis in *A. nidulans*.

### 3.3. Schizzosaccharomyces pombe

The yeast *S. pombe* undergoes a different pattern of morphogenesis from *S. cerevisiae* in that it grows as a rod-shaped cell that divides by fission. Three proteins related to ScPil1/Lsp1are detected in *S. pombe*: Pil1, Pil2 and the more distantly related Meu14 protein. Although Pil1 and Pil2 share 46% identity, they have distinct patterns of expression and localization. *S. pombe* Pil1 is detected in the PM of both vegetative and mating cells, whereas Pil2 is only expressed in mating cells and localizes to the PM of the meiotic spores that develop after conjugation [13,14]. Interestingly, Pil1 localizes to patches in the PM that are about 1 μm, which makes them five-fold longer than the eisosomes in *S. cerevisiae*. These eisosomes did not show a uniform orientation relative to the cell growth axis. The formation of the longer eisosomes appears to be a product of the cellular environment and is not an intrinsic property of the *S. pombe* Pil1 proteins [13]. Expression of *S. cerevisiae* Pil1 (*Sc*Pil1) in *pil1Δ pil2Δ S. pombe* mutant cells resulted in the formation of long filaments of *Sc*Pil1-GFP at the cell cortex rather than the shorter eisosome structures seen in *S. cerevisiae* cells. Conversely, *S. pombe* Pil1 (*Sp*Pil1) expressed in *pil1Δ S. cerevisiae* cells co-localized with ScLsp1 to small puncta at the cell cortex that were the size expected for *S. cerevisiae* eisosomes. Deletion of *S. pombe pil1* did not result in an obvious morphological phenotype, but its overexpression resulted in the formation of long cytoplasmic Pil1 filaments and impaired morphogenesis and cytokinesis [13]. The third Pil1/Lsp1-related protein in *S. pombe*, Meu14, was upregulated during meiosis and localized to ring structures in the spore membrane that did not appear to colocalize with eisosome filaments [56].

Analysis of other MCC/eisosome proteins in *S. pombe* showed that the Nce102 related tetraspanner protein Fhn1 colocalized with Pil1, and studies of the corresponding deletion mutants showed that their localization was interdependent [13]. This is similar to results for Nce102 and Pil1 in *S. cerevisiae* but distinct from *A. gossypii. S. pombe* Pil1 may also be regulated by phosphorylation similar to *S. cerevisiae* Pil1. SpPil1 was found in a high throughput study to be phosphorylated on several sites, including Thr-230 and Thr-233 [57], which correspond to phosphorylated sites in *S. cerevisiae* Pil1 that have been shown to be functionally important for regulation of eisosome formation [26,27]. Surprisingly, some MCC/eisosome proteins did not co-localize with Pil1 as expected. Fluorescent protein fusions to Sur7 and Slm1 both appeared to primarily localize to the growing cell ends rather than the eisosomes in the central region [13]. Interestingly, another tetraspanner protein, Mug33, also localized to the ends of cells, but a truncated version lacking the C-terminal tail colocalized with Pil1 in eisosomes [14]. Thus, it remains to be determined if the failure of *S. pombe* Sur7-GFP to colocalize with eisosomes is due to its altered C terminus, or if it is an intrinsic property of the wild-type Sur7 protein. C terminal sequences are also important for proper localization of the tetraspanner protein Nce102 in *S. cerevisiae* [42].

*S. pombe* is similar to *S. cerevisiae* and other fungi in that eisosomes are not detected at sites of new cell growth. Moreover, an interesting feature of the fission mode of replication for *S. pombe* is that eisosomes were cleared away from the future zone of cell division that eventually forms across the middle of the cell [13]. During the start of the cell cycle, eisosomes are detected in the mid region of the cells, but not at the growing tips. Then, during the late stages of the cell cycle, the eisosomes disappear from the mid region of the cell that corresponds to the future site of cytokinesis. Their removal appears to be due to a combination of disassembly and also movement out of the central zone [13]. This is the first reported observation of eisosome movement in any organism.

## 4. MCC Eisosomes in the Human Pathogen *Candida albicans*

The human fungal pathogen *Candida albicans* commonly exists as a harmless commensal organism on the skin and gastrointestinal tract of humans. Medical interventions or immunosuppression permit *C. albicans* to enter the bloodstream and invade tissues, which can lead to organ failure and death [58,59]. Changes in medical care are leading to increased candidiasis and the lack of efficient antifungal drugs makes these infections difficult to treat [60]. Thus, a better understanding of the mechanisms that permit survival in the host is needed to develop novel therapeutic approaches. In particular, knowledge of the PM is limited, although it plays a multi-faceted role in *C. albicans* pathogenesis by mediating environmental sensing, nutrient uptake, virulence factor secretion, cellular morphogenesis, and cell wall biogenesis. The significance of studies on the PM is highlighted by the fact that the most effective antifungal drugs currently used target lipids in this essential barrier or the resident proteins [1].

### 4.1. MCC/Eisosome Proteins in *C. albicans*

*C. albicans* can grow as a budding yeast similar to *S. cerevisiae*, and these organisms share many common characteristics. However, they also have very distinct features, such as the ability of *C. albicans* to switch to hyphal morphogenesis under conditions that mimic infection of a human host [61]. On a molecular level they are also distinct, as the median protein identity between these species is only about 50% [62]. Consistent with this, there are many similarities in their MCC/eisosome proteins and there are also some critical differences. In particular, some orthologs of *S. cerevisiae* MCC proteins are absent in *C. albicans*. For example, the Sur7 paralogs Pun1 and Ynl194c are not readily detected by homology searches in *C. albicans*. Similarly, although orthologs of Pil1, Lsp1, Pkh1 and other eisosome proteins are present, some redundant proteins, such as Pkh2, are not detected. Nonetheless, Sur7-GFP, Fmp45-GFP, Lsp1-GFP and Pil1-GFP all localize to stable punctate patches in the PM that are generally similar to those seen for *S. cerevisiae* MCC/eisosomes [17,48,63,64].

### 4.2. Sur7 is Needed for Proper Morphogenesis and Cell Wall Synthesis

In contrast to *S. cerevisiae*, a *C. albicans sur7Δ* mutant displays strong defects in morphogenesis and cell wall synthesis [17,63,64]. Morphogenesis was very abnormal, including irregularly shaped buds and a defect in septation. The *sur7Δ* mutant is also defective in switching to hyphal growth, frequently forming broader hyphal structures rather than thin filamentous hyphal cells. This morphogenesis defect also correlates with a defect in invasive growth of hyphal filaments into agar and to a defect in biofilm formation [17,64]. One of the most unique defects of the *sur7Δ* mutant is that it formed long projections of cell wall growth into the cytoplasm [17]. These intracellular projections of cell wall growth are much more extreme than the irregularities in the cell wall that are observed in an *S. cerevisiae pil1Δ* mutant [6].

Further analysis showed that the *sur7Δ* mutant cells not only form abnormally shaped cell walls, but also that the cell walls are defective. The *sur7Δ* cells are more sensitive to factors that exacerbate cell wall defects, such as detergent (SDS) and the chitin-binding agent Calcofluor White [63]. The *sur7Δ* cells are also slightly more sensitive to inhibitors that block the synthesis of cell wall chitin (nikkomycin Z) and β-1,3 glucan (caspofungin). Interestingly, the *sur7Δ* cells are 8-fold more sensitive to the drug cercosporamide that inhibits Pkc1 from inducing cell wall repair genes [63]. It appears that the Pkc1 pathway is active in *sur7Δ* cells, but that there is a higher requirement for its function to compensate for the cell wall defects that occur under normal growth conditions. Microarray analysis of gene expression showed that many cell wall synthesis genes were highly induced in *sur7Δ* cells grown under standard conditions. Interestingly, the pattern of genes induced in *sur7Δ* cells showed many similarities to that seen for cells that were treated with caspofungin to inhibit cell wall β-glucan synthase [17].

Chemical analysis of cell wall composition demonstrated that *sur7Δ* cells contain decreased levels of β-glucan, a glucose polymer that confers rigidity to the cell wall [63]. Consistent with this, *sur7Δ* cells were more sensitive to lysis, which could be partially rescued by increasing the osmolarity of the medium. Interestingly, Sur7 is present in static patches, whereas β-1,3-glucan synthase is mobile in the PM and often associated with actin patches [65,66,67]. Thus, Sur7 may influence β-glucan synthesis indirectly, perhaps by altering the function of the cell signaling components that localize to the MCC and eisosome domains.

### 4.3. Sur7 is Needed for Proper PM Organization

The *C. albicans sur7Δ* mutant displays altered PM organization that is likely to contribute to the other defects seen for this mutant. In particular, two key landmark proteins are altered in the *sur7Δ* mutant: actin and septins [17]. Actin localization is diffuse and actin patches are no longer highly polarized to sites of active morphogenesis at the tips of buds and hyphae, consistent with the observed defects in morphogenesis. In addition, septins are mislocalized to ectopic sites away from the bud neck in the *sur7Δ* mutant. Septin localization in wild-type cells is tightly restricted to the bud neck, where the septins form a ring on the inner surface of the PM that carries out several functions [68,69]. One is to act as a barrier that restricts actin patches and PM proteins to the bud [70]. Thus, abnormal septin ring function could help contribute to the mislocalization of actin seen in *sur7Δ* mutants. Another important septin function is to recruit chitin synthase to the bud neck. Thus, it is possible that the mislocalized septins present at ectopic sites could help to nucleate cell wall growth at these sites away from the bud neck. In support of the link between altered septin localization and intracellular wall growth, certain *S. cerevisiae* septin mutants and a *cla4Δ* mutant, which lacks a regulator of septins, display ectopic cell wall growth that is similar to some of the abnormal wall growth seen in the *sur7Δ* mutant [71,72]. Lsp1-GFP still localized to punctate patches in the *sur7Δ* mutant, indicating that the defects described above are not due to the failure to form eisosomes.

### 4.4. Sur7Δ Defects in Virulence Functions

Consistent with the diverse phenotypes caused by deletion of *SUR7*, *sur7Δ* mutants are defective in several different virulence-related functions [17,63,64,73]. The *sur7Δ* cells show defects in hyphal morphogenesis and invasive growth into agar, which are important functions for mediating invasive growth into tissues. The *sur7Δ* mutant is also defective in biofilm formation, secretion of lipase, and in macrophage killing [64]. These phenotypes likely contribute to the strong virulence defect in a mouse model of systemic *C. albicans* infection [73].

### 4.5. Implications for Antifungal Therapy

The PM and its resident proteins are directly or indirectly the targets of most of the commonly used antifungal drugs [1]. Studies on MCC/eisosome domains are therefore expected to lead to a better understanding of the mechanisms by which these drugs act. For example, MCC domains preferentially stain with the ergosterol-binding agent filipin [19], suggesting that the related drug amphotericin may preferentially bind to MCC domains. Also, *sur7Δ* cells are 5-fold more sensitive to fluconazole, an inhibitor of ergosterol synthesis [17]. The *sur7Δ* cells are ∼2-fold more sensitive to caspofungin, which inhibits the formation of β-glucan, the major component of the cell wall. This correlates with altered cell wall formation and decreased cell wall β-glucan in *sur7Δ* mutant cells [63,64]. The cell wall defects of *sur7Δ* cells are also thought to account for the 8-fold increased sensitivity to the drug cercosporamide that prevents the Pkc1 protein kinase from inducing cell wall repair genes [63]. Studies on Sur7 and other MCC/eisosome components also have important implications for the development of novel therapeutic approaches. An advantage of targeting MCC domains is that inhibitory drugs are expected to cause multiple defects in PM organization that impact a wide range of virulence factors rather than blocking one specific function. The MCC/eisosome components are also interesting drug targets in that they are not highly conserved in mammalian cells. Thus, further studies on MCC/eisosome domains will be important to help to make current drug therapy more effective and to reveal new avenues for therapeutic intervention.

## 5. Future Outlook

The discovery and characterization of MCC/eisosome domains has rapidly progressed over the last few years. Future studies will build on this foundation to further define the mechanisms that regulate MCC/eisosome assembly and disassembly. These studies will also create tools to help define in more detail the biological roles of MCC/eisosomes in PM organization. For example, it will be important to address the question of how the immobile MCC/eisosomes can affect the localization and organization of actin and the cell wall synthesis proteins that reside outside of these domains. It will also be important to define the functional roles of MCC/eisosomes. Are they acting only as scaffolds to promote proper PM organization? Or do they have other functions, such as sensing PM stress or contributing to lipid homeostasis? A better understanding of the mechanisms of MCC/eisosome function will also serve as a model to provide insight into the role of PM organization in mammalian cells. Although the MCC/eisosome proteins are not highly conserved outside of the fungal kingdom, they share fundamental similarity with membrane proteins in mammalian cells. For example, mammalian cells contain BAR domain family proteins similar to Pil1, Lsp1, and Slm1/2 [29]. Also, Sur7 and Nce102 share interesting similarity to mammalian tetraspanner proteins that play key roles in PM organization, including Claudin proteins at tight junctions and Tetraspanin-enriched domains in lymphocytes [17,74,75,76]. Identification of the structure and function of these protein-organized domains in different organisms will hopefully synergize to promote a better understanding of PM function.
